# Giant cell tumor stromal cells: osteoblast lineage-derived cells secrete IL-6 and IL-10 for M2 macrophages polarization

**DOI:** 10.7717/peerj.9748

**Published:** 2020-08-24

**Authors:** Kuan Yang, Lihui Bao, Xiaoning He, Wanmin Zhao, Dongdong Fei, Bei Li, Yang Xue, Zhiwei Dong

**Affiliations:** 1 State Key Laboratory of Military Stomatology & National Clinical Research Center for Oral Diseases & Shaanxi International Joint Research Center for Oral Diseases, Center for Tissue Engineering, School of Stomatology, The Fourth Military Medical University, Xi’an, Shaanxi, China; 2 Xi’an Institute of Tissue Engineering and Regenerative Medicine, Xi’an, Shaanxi, China; 3 State Key Laboratory of Military Stomatology & National Clinical Research Center for Oral Diseases & Shaanxi Clinical Research Center for Oral Diseases, Department of Oral and Maxillofacial Surgery, School of Stomatology, The Fourth Military Medical University, Xi’an, Shaanxi, China

**Keywords:** Giant cell tumor stromal cells, Macrophages, Polarization, Interleukin-6, Interleukin-10

## Abstract

**Background:**

The giant cell tumor (GCT) is a benign tumor which consists of three types cells: mononuclear histiocytic cells (MNHCs), multinuclear giant cells (MNGCs), and GCT stromal cells (GCTSCs). Numerous studies claim that GCTSCs have mesenchymal stem cells (MSCs) characters and play an important role in osteoclastogenesis; however, there are no research studies concerning macrophage polarization among GCT, which can be regarded as an ingredient for tumor aggression.

**Method:**

We tested the effect of GCTSCs from three GCT samples which were collected from patients on proliferation, apoptosis and polarization of macrophage.

**Result:**

In this article, we verified that GCTSCs expressed MSCs markers and had higher proliferation and relative lower differentiation abilities compared with BMMSCs. What’s more, we found a higher proportion of M2 macrophages among neoplasm. Co-culturing GCTSCs with macrophages resulted in prominent macrophage M2 polarization and increased the release of IL-6 (Interleukin-6) and IL-10 (Interleukin-10)from GCTSCs. In conclusion, GCTSCs, as originating from MSCs, can secret IL-6 and IL-10, which may play a significant role in macrophage M2 polarization.

## Introduction

The giant cell tumor (GCT) is a locally aggressive benign tumor which incidence accounts for nearly 3–5% of primary bone neoplasm (Thomas & Skubitz, 2009). About 80% patients with GCT are aged 20 to 40. The GCT induced lesions usually occur at long bones, mostly located at distal femur, proximal tibia, and distal radius, sometimes at sacrum. The typical radiographic features of GCT include lytic mass with cystic degeneration and intact fibrous periosteum margin (Raskin et al., 2013; Chakarun et al., 2013).

GCT consists of three mainly cells types: the mononuclear histiocytic cells (MNHCs), which are driven from hematopoietic system, multinuclear giant cells (MNGCs), and GCT stromal cells (GCTSCs). GCTSCs are the neoplastic component of GCT, which is regarded as a promoter for accumulation, size and activity of giant cells (Wuelling, Delling & Kaiser, 2004; Werner, 2006; Zheng et al., 2001). It has been claimed that GCTSCs originate from mesenchymal stem cells (MSCs) (Werner, 2006; Wülling, Delling & Haiser, 2003) and express MSCs markers CD73, CD105, CD166 (Wülling, Delling & Haiser, 2003). What’s more, GCTSCs are also capable of multilineage differentiation into adipocytes and osteocytes, which can secret bone matrix proteins such as type I collagen, osteocalcin and bone sialoprotein (Wülling, Delling & Haiser, 2003; Robinson, Segal & Nevo, 2002; Lan et al., 2012; Murata et al., 2005).

However, apart from the above features, the mechanisms that how GCTSCs affect tumor cell invasion and metastasis are still unclear. In view of GCT neoplasm is full filled with macrophages, we speculate GCTSCs may have a connection with tumor associated macrophages (TAMs) (Kim & Bae, 2016). It has been evidenced that transplanting MSCs into experimental spinal cord injury model alternatively activated M2 macrophages (Nakajima et al., 2012). As an anti-inflammatory factor, M2 macrophages regulate inflammatory responses and promote angiogenesis, tissue remodeling and repair, which enable them to play a significant role in tumor aggression (Mantovani et al., 2002; Ohashi et al., 2018).

Some studies have demonstrated that MSCs can convert macrophages into M2 phenotype by producing IL-10 (Hutchins, Poulain & Miranda-Saavedra, 2012), which has been evidenced as major cytokine inducing M2 polarization (Murray, 2017; Jiang et al., 2016; Mauer et al., 2014; Goswami et al., 2017). In the current study, we analyzed the expression of cytokines in BMMSCs and GCTSCs. The result showed an enhanced secretion of IL-6 and IL-10 in GCTSCs while co-cultured with macrophages. Considering GCTSCs originate from MSCs, it has been hypothesized that GCTSCs might influence M2 polarization of the macrophages in which IL-6 and IL-10 play an important role. In this study, we showed that GCTSCs promoted macrophages M2 polarization via increased IL-6 and IL-10 secreting, which resulted in GCT protruding aggression.

## Materials & Methods

### Clinical samples

Three GCT tissue samples were collected from patients who underwent surgical resection for primary GCT in Department of Orthopaedics, Xijing Hospital, the Fourth Military Medical University. The patient’s consent was obtained and informed consent form was signed. The use of all patient-derived materials was approved by the Ethics Committee of the Fourth Military Medical University (IRB-REV-2015005), and patient informed consent was obtained individually.

### Cell culture

Primary GCT stromal cells (GCTSCs) were isolated from fresh GCT. The neoplasm was minced in small pieces and digested with 200 U/ml collagenase (Gibco BRL, Karlsruhe, Germany) for 1 h at 37 °C. Cells were collected by centrifugation, washed twice in PBS and cultured with alpha minimum essential medium (α-MEM, Gibco BRL, Gaithersburg, MD, USA) containing 10% FBS (Hangzhou Sijiqing Biological Engineering Materials Co., Ltd. Zhejiang, China) and 100 U/ml penicillin and 100 mg/ml streptomycin (Invitrogen Life Technology, Carlsbad, CA, USA). The cells were kept in culture at 37 °C, 5% CO_2_, and the media was changed every 3 days thereafter until cells reached 70% confluency. Adherent GCTSCs were harvested using 0.25% Trypsin-EDTA (Invitrogen Life Technology, Carlsbad, CA, USA). Normal MSCs were obtained from the bone marrow of the normal donor patients as described previously (Soleimani and Nadri 2009). Human monocytes were isolated from the peripheral blood of normal human volunteers (Blood donors in Blood Transfusion Department of Xijing Hospital) using human monocyte isolation kit II (Miltenyi Biotech, Teterow, Germany). Purified CD14+ monocytes were plated into 6-well cell culture plates at a concentration of 1 × 106 per well in RPMI 1,640 media supplemented with 10% FBS.

### Animal experiments

Immunocompromised mice (NMRI-nu/nu) were purchased from Beijing Vital River Laboratory, Beijing, China. All mice were housed five per cage under standard laboratory conditions at 12 h light /dark cycles, and 22 °C, with free access to food and water. Mice were anesthetized by intraperitoneal injection of pentobarbital (30 mg/kg) and implanted with cells. After 35 days of implantation, the mice were decapitated and killed, the de novo bone area was taken. Animal experiments in this study was authorized by the Animal Care Committee of Fourth Military Medical University (2016-kq-054), and all experimental protocols were performed with the approval of the Fourth Military Medical University.

### Histology and immunohistochemistry staining

The neoplasm specimens were fixed in 4% paraformaldehyde overnight and then embedded in paraffin or in the optimal cutting temperature compound. Hematoxylin and eosin (HE) staining was used for general observation. Surface markers of GCTSCs and macrophages were stained with CD105 and CD14 antibodies (eBiosciences, San Diego, CA, USA) overnight at 4 °C, respectively. When they were washed 3 times, peroxidase conjugated goat anti-rabbit secondary antibody (Dako, Denmark) was added. Envision Detection System kit (DAKO, Denmark) was used after the nuclear stained by hematoxylin. Color development was triggered using Di-Amino Benzidine (DAB, Calbiochem, Germany).

### Immunofluorescent staining

BMMSCs and GCTSCs were washed with PBS and fixed with 4% paraformaldehyde for 15 min, and then were incubated with antibodies CD146, CD90, CD73, STRO-1 and SSEA-4, as well as antibodies against hematopoietic cell markers of CD14 and CD34 and CD45 (All from BD Biosciences, San Diego, CA, USA). The positively stained cells were detected by a laser scanning confocal microscope (Olympus FluoView FV 1000, Tokyo, Japan).

### Flow cytometry analysis

To confirm mesenchymal stem cells character, we used flow cytometric analysis to show that GCTSCs were positive for CD90, CD105, and CD29, and negative for CD14, CD34 and CD45 (All from BD Biosciences, San Diego, CA, USA). Ki-67 staining was used to confirm the proliferation of macrophages after being co-cultured with GCTSCs. Apoptotic cells were examined using Annexin V-FITC/7AAD Apoptosis Detection Kit (Roche, Basel, Switzerland). Cells were stained with Annexin V-FITC and 7AAD for 15 min, both of which were in the dark at room temperature. After washing by PBS, immunofluorescent-stained cells were analyzed on FACS (Becton-Dickinson) can using Consort 30 software.

### BrdU test

BMMSCs and GCTSCs were labeled with 5-bromodeoxyuridine (BrdU, sigma) 10 umol L^−1^ for 24 h. After the cells were washed three times with PBS and fixed with 4% paraformaldehyde for 15 min, the incorporated BrdU was detected by mouse anti-BrdU monoclonal antibody conjugated with peroxidase (Abcam). The color reaction was parallel with the number of proliferating cells.

### Osteogenic and adipogenic differentiation

BMMSCs and GCTSCs were incubated with osteogenic medium (100 nmol L^−1^ dexamethasone, 50 mg mL^−1^ ascorbic acid and one mmol L^−1^ b-glycerophosphate, Sigma) for 21 d according to the manufacturer’s instructions. Cells were fixed with 60% isopropanol and stained with 1% Alizarin Red (Sigma) for osteogenic differentiation assay. BMMSCs and GCTSCs were also cultured with adipogenic medium (0.5 mmol L^−1^ methylisobutylxanthine, 0.5 mmol L^−1^ hydrocortisone and 60 mmol L^−1^ indomethacin, Sigma) for 14 d. Intracellular lipid droplet was detected by staining with Oil Red O solution.

### In vivo bone formation assay

5 × 10^6^ cells were combined with 40 mg hydroxyapatite/tricalcium phosphate (HA/TCP) and implanted at the back of immunocompromised mice (NMRI-nu/nu). After 35 days the mice were euthanized and the de novo bone area was stained by HE. The sections were analyzed by Image-Pro Plus software.

### Co-cultures

The purified human blood monocytes were indirectly co-cultured with GCTSCs as well as BMMSCs for 14 d. The monocytes (2 ×10^5^ cells) were cultured in 24 well plates and GCTSCs or BMMSCs (2 ×10^4^ cells) were cultured in the cell-culture insert (pore size: 0.4 pm) of the same well (Falcon Inc.).

### Antibody-based protein array

The assay was performed following the manufacturer’s instructions (RayBio). Briefly, samples with equal amounts of total proteins were incubated overnight at 4 °C. After washing, the membranes were incubated with Biotinylated Antibody Cocktail followed by second wash. Incubate with HRP-Streptavidin overnight at 4 °C and wash again. Chemiluminescence was used for signal detection, which was analyzed with Quantity One image analysis software (Molecular Imager FX, Bio-Rad Laboratories).

### Quantitative RT-PCR

Total RNA was isolated from BMMSCs and GCTSCs by using RNAiso plus (TaKaRa, Tokyo, Japan) according to the manufacturer’s instructions. The mRNA was reversed to complementary DNA, and quantitative real time -PCR (qRT-PCR) detection was carried out by Prime Script TM RT master mix (TaKaRa, RR036A) and SYBR Premix Ex TaqTMII (TaKaRa). A CFX96 Trademark Real-time PCR detection system (Bio-Rad, Richmond, CA, USA) was used for the detection. The forward and reverse primers were used to measure IL-6 (F: AGTGAGGAACAAGCCAGAGC,R: AGCTGCGCAGAATGAGATGA) and IL-10 (F: ACCTGCCTAACATGCTTCGAG, R: TGGGTCTTGGTTCTCAGCTTG) gene expression, and target gene expression levels were normalized to that of GAPDH (F: TGACATCAAGAAGGTGGTGAAGC, R: GGAAGAATGGGAGTTGCTGTTG).

### Gene ablation by siRNA

2 × 10^5^ cells/well were seeded in a 6-well culture plate with 2 ml antibiotic- free normal growth medium supplemented with FBS. Incubated the cells at 37 °C in a CO2 incubator until the cells were 60–80% confluent. Then added IL-6 (Santa) and IL-10 (Santa) siRNA duplex and siRNA transfection reagent (Santa) mixture after cells were washed with two mL siRNA transfection medium, then incubate the cells 5–7 h at 37 °C in a CO_2_ incubator. one mL of medium containing 2 times FBS and antibiotics was added to cells and incubated for additional 18–24 h before used.

### Statistical analysis

GraphPad Prism software (Graph-Pad Software, Inc., USA) was used for statistical analysis. All data are presented as mean ± s.d and the measurements were performed on three different GCT patient samples (*n* = 3). Unpaired, two-tailed Student’s t tests for comparisons between two groups and one-way analysis of variance (ANOVA) with Bonferroni for multiple comparisons were applied. Each experiment was repeated at least three times. *P* value < 0.05 was considered to be statistically significant.

## Results

### CD14 and CD105 cells exist in Gaint cell tumor

**Figure 1:**
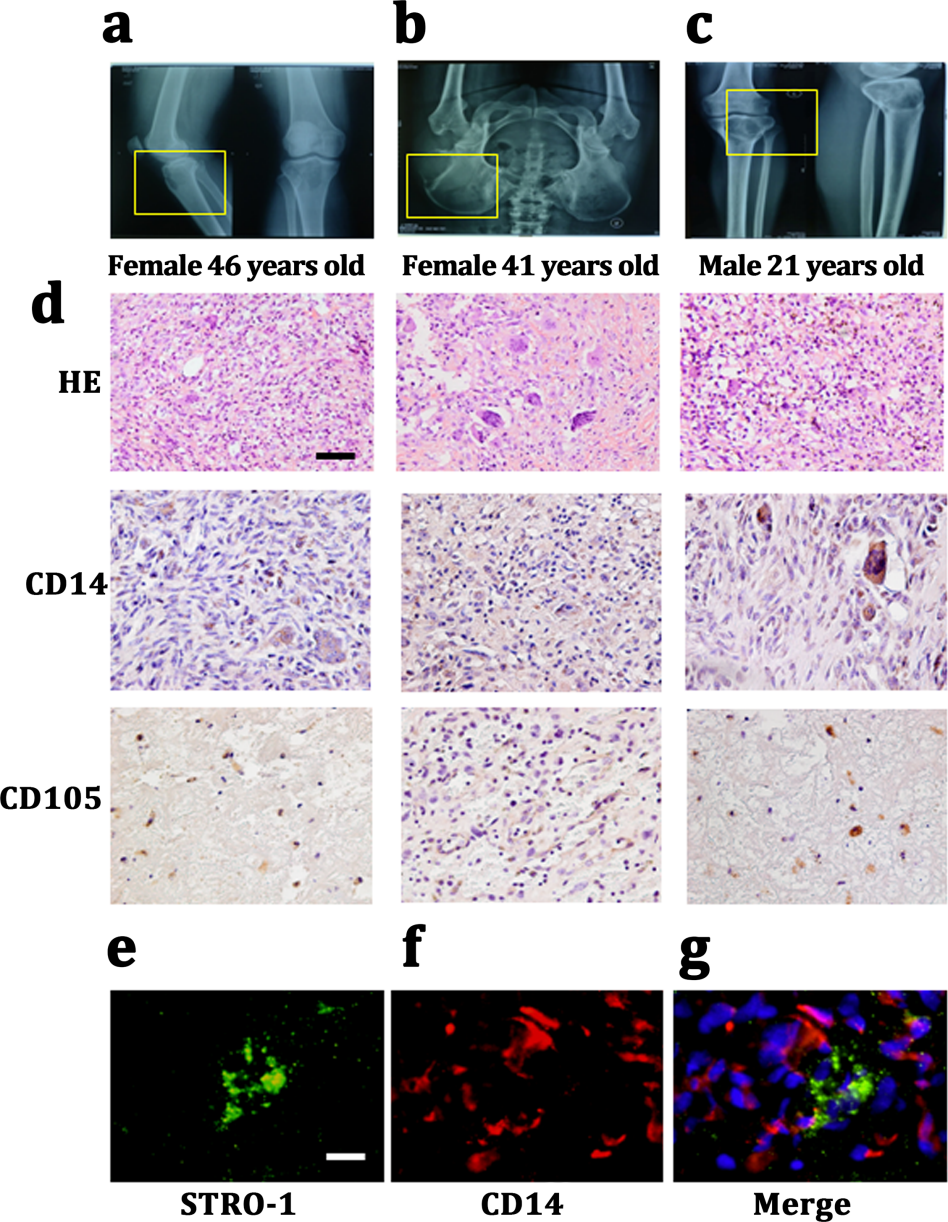
CD14 and CD105 cells are observed in Gaint cell tumor by tissue staining. (A–C) X-Rays indicating the lesion area of three GCT patients are tibia, ilium and both terminal of the tibia and femur, respectively. (D) HE staining indicates that the neoplasm is consist of three categories cells: multinucleated giant cells, mononuclear histiocytic cells and GCTSCs. Scale bar = 50 µm. (E–G) Immunofluorescent staining exhibits the STRO-1^+^ cells (green) have a different distribution with CD14^+^ cells (red), which means that they belong to two distinguished lineages. Scale bar = 10 µm.

We harvested Giant cell tumor (GCT) samples from three patients with two females (46 and 41 years old) and one male (21 years old). The lesions in which were tibia, ilium and both terminal of the tibia and femur. All of these three patients presented a common radiologic feature: a lytic and well-defined lesion without sclerotic margin (Figs. 1A– 1C). HE staining showed GCT consisted of three kinds of cells: multinucleated giant cells, mononuclear histocytic cells and giant cell tumor stromal cells (GCTSCs) with a spindle morphology. Immunohistochemistry staining verified that GCT cells contained CD14 positive and CD105 positive cells, which represented a macrophage-like and mesenchymal-like features respectively (Fig. 1D). Immunofluorescence staining of the GCT indicated STRO-1 positive and CD14 positive cells in the samples and these two kinds of cells were not overlapped (Figs. 1E, 1F).

### GCTSCs showed increased proliferation and decreased differentiation characteristics

**Figure 2:**
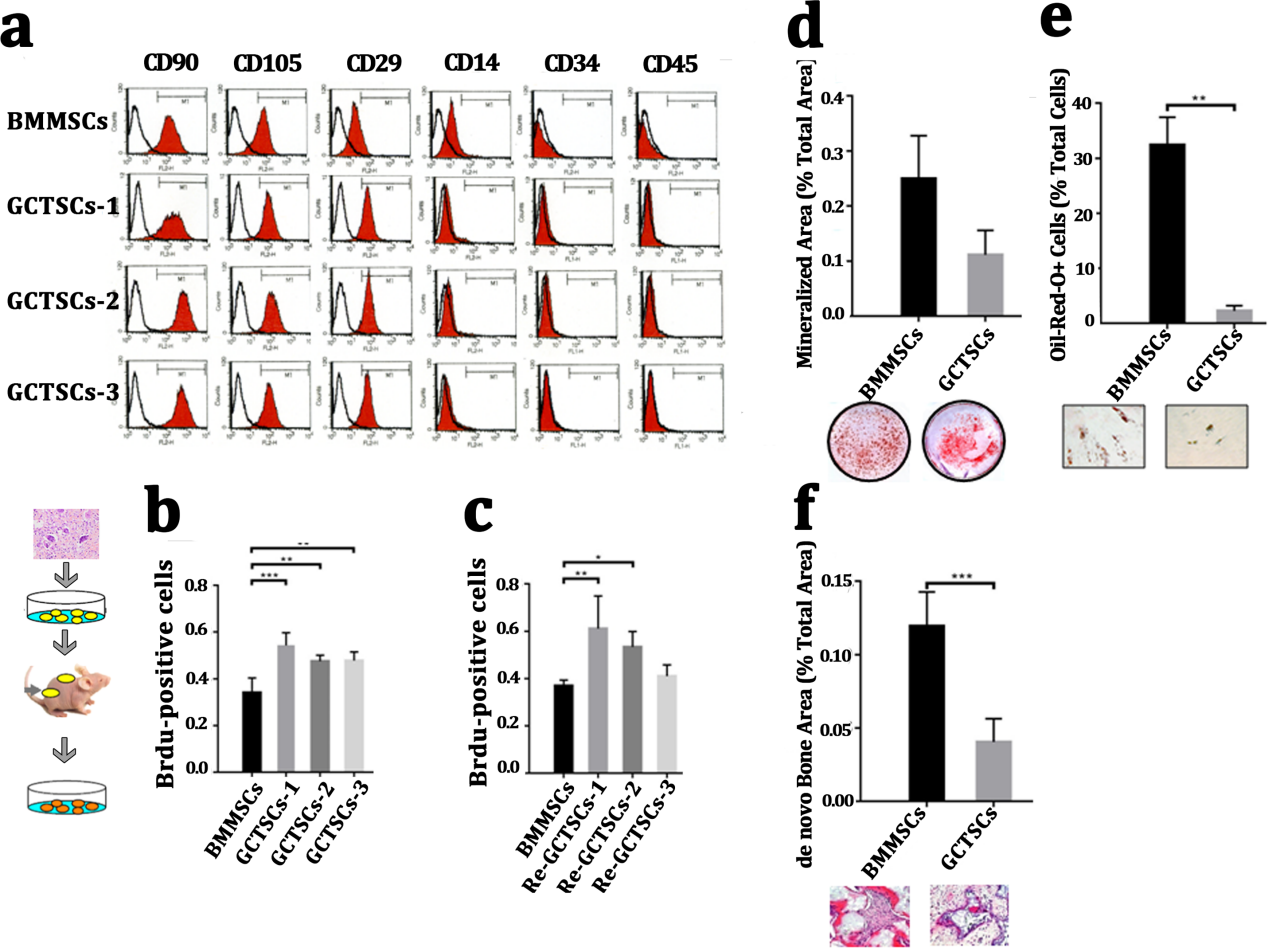
GCTSCs showed increased proliferation and decreased differentiation characteristics. (A) Flow cytometry analysis indicates that both of BMMSCs and GCTSC1, GCTSC2, GCTSC3 have common expressed MSCs markers CD90, CD105, CD29, and rarely express hematopoietic lineage markers CD14, CD34, CD45. (B, C) Proliferation ability of both BMMSCs and GCTSCs. Brdu measurement indicates the proliferating cells. Single-colony-derived MSCs and GCTSCs, respectively, were expanded to 2 ×10^6^ cells and subsequently implanted into immunocompromised mice with the use of geofoams as a carrier. The proliferating cells in GCTSCs1 (*P* < 0.01) and GCTSCs2 (*P* < 0.05) are much more than BMMSCs. However, the GCTSCs3 after implantation shows a similar proliferation ability. (D, E) Osteogenic and adipogenic differentiation of BMMSCs and GCTSCs in vitro. (F) *de novo* bone formation of BMMSCs and GCTSCs in vivo.

It had been widely proved that giant cell tumor stromal cells (GCTSCs) were the neoplastic component of GCT and had similar functions with mesenchymal stem cells. We isolated the cells from neoplasm and compared different bio-markers (CD90, CD105, CD29, CD14, CD34, CD45) between three patients’ GCTSCs and normal bone marrow MSCs. Flow cytometer analysis indicated that both GCTSCs and BMMSCs had a similar specific expression of CD90, CD105 and CD29 and negative expression of CD14, CD34 or CD45 (Fig. 2A). To characterize the proliferation property of GCTSCs, we performed serial implantation of GCTSCs. Brdu measurement indicated that all of three GCTSCs had a significantly higher proportion of Brdu-positive cells than BMMSCs (*P* < 0.01). After subcutaneously implantation of GCTSCs into immunocompromised mice, we found that re-GCTSCs collected from implants still showed higher proliferation than re-BMMSCs. (Figs. 2B, 2C). These results indicated GCTSCs had mesenchymal stem cell like properties and had an obviously higher proliferative activity than BMMSCs.

In order to examine the differentiative capacity of GCTSCs into osteoblast and adipocyte, we induced the GCTSCs with osteogenic and adipogenic induction medium respectively. Alizarin Red staining verified that the total mineralized area in GCTSCs was 0.11 compared with 0.25 in BMMSCs (2.27-fold higher, *P* = 0.056) (Fig. 2D). Oil-Red O stained cells represented the adipocytes, the proportion of which in GCTSCs was 2.22% compared with 32.44% in BMMSCs (14.61-fold higher, *P* < 0.01) (Fig. 2E). GCTSCs or BMMSCs combined HA/TCP were subcutaneously implanted into immunocompromised mice, and a clearly bone formation area of BMMSC implantation was founded, the total *de novo* bone area was about 0.12%, compared with much smaller spots about 0.04% among GCTSC implantation (*P* < 0.001) (Fig. 2F). These results indicated that GCTSCs indeed had bone and adipose differentiation capacities to some extent in vitro as well as in vivo, but which were much weaker than BMMSCs.

### GCTSCs play no role in proliferation or apoptosis of CD14 cells

**Figure 3:**
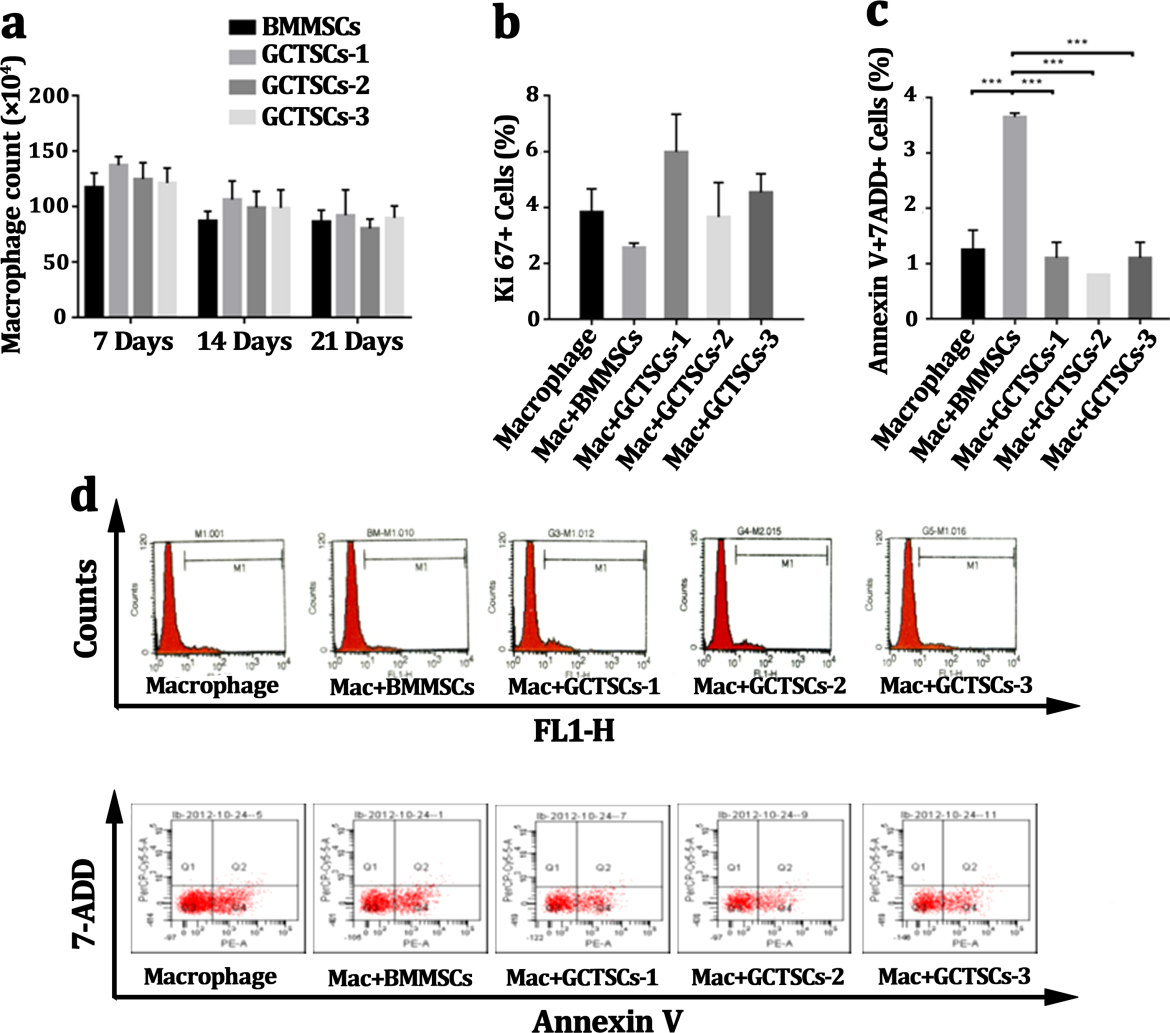
GCTSCs play no role in proliferation or apoptosis of CD14 cells. (A) The macrophages number co-cultured with BMMSCs and GCTSCs were compared in 7, 14 and 21 days. It shows that the both BMMSCs and GCTSCs have a rare effect on macrophages cells number. (B) Ki67 expression in macrophages co-culturing with different types of cells shows a similar level (*P* > 0.05), which indicates that both BMMSCs and GCTSCs don’t have a promotion for macrophages proliferation. (C) The apoptosis of macrophages co-culturing with different types of cells shows that BMMSCs (*P* < 0.001) have a prominent tendence to induce macrophages apoptosis, compared with GCTSCs (*P* > 0.05). (D) Representative flow cytometry analysis of macrophages proliferation and apoptosis co-cultured with BMMSCs and GCTSCs.

Many researches had pronounced that GCTSCs played a significant role in giant cells formation, osteolysis as well as aggression, but there were rarely researches to explore the influence of GCTSCs on the proliferation or apoptosis of CD14 cell. Co-culturing macrophage cells with GCTSCs and BMMSCs using transwell for 7 days, 14 days and 21 days respectively and the amount of macrophage cells was counted. The results of different co-culturing period with GCTSCs or BMMSCs exhibited no significance correlation (Fig. 3A). The proportion of Ki67 cells, as an indication of proliferative ability, among normal macrophages and co-cultured with BMMSCs as well as GCTSCs are 3.83%, 2.57% and 4.72% (*P* > 0.05) (Figs. 3B, 3D), which indicated both GCTSCs and BMMSCs didn’t alter the proliferative ability of CD 14 positive macrophages.

Moreover, we detected the Annexin V+7-ADD+ macrophages after co-cultured with BMMSCs or GCTSCs. BMMSCs had a prominent tendency in inducing macrophage apoptosis, the proportion of which was approximately 3.65% (*P* = 0.01), however, co-culture with GCTSCs hadn’t resulted in any enhancement of macrophage apoptosis, which were 1.25%, 3.65% and 1% (BMMSCs vs Macrophage, *P* < 0.05. GCTSCs vs Macrophage, *P* > 0.05), respectively (Figs. 3C, 3D). These data confirmed that BMMSCs could induce the apoptosis of macrophage but not the GCTSCs. However, the proportion of apoptosis cells induced by BMMSCs was very low.

### GCTSCs promote the polarization into M2 phenotype of macrophage via increasing IL-6 and IL-10 release

**Figure 4:**
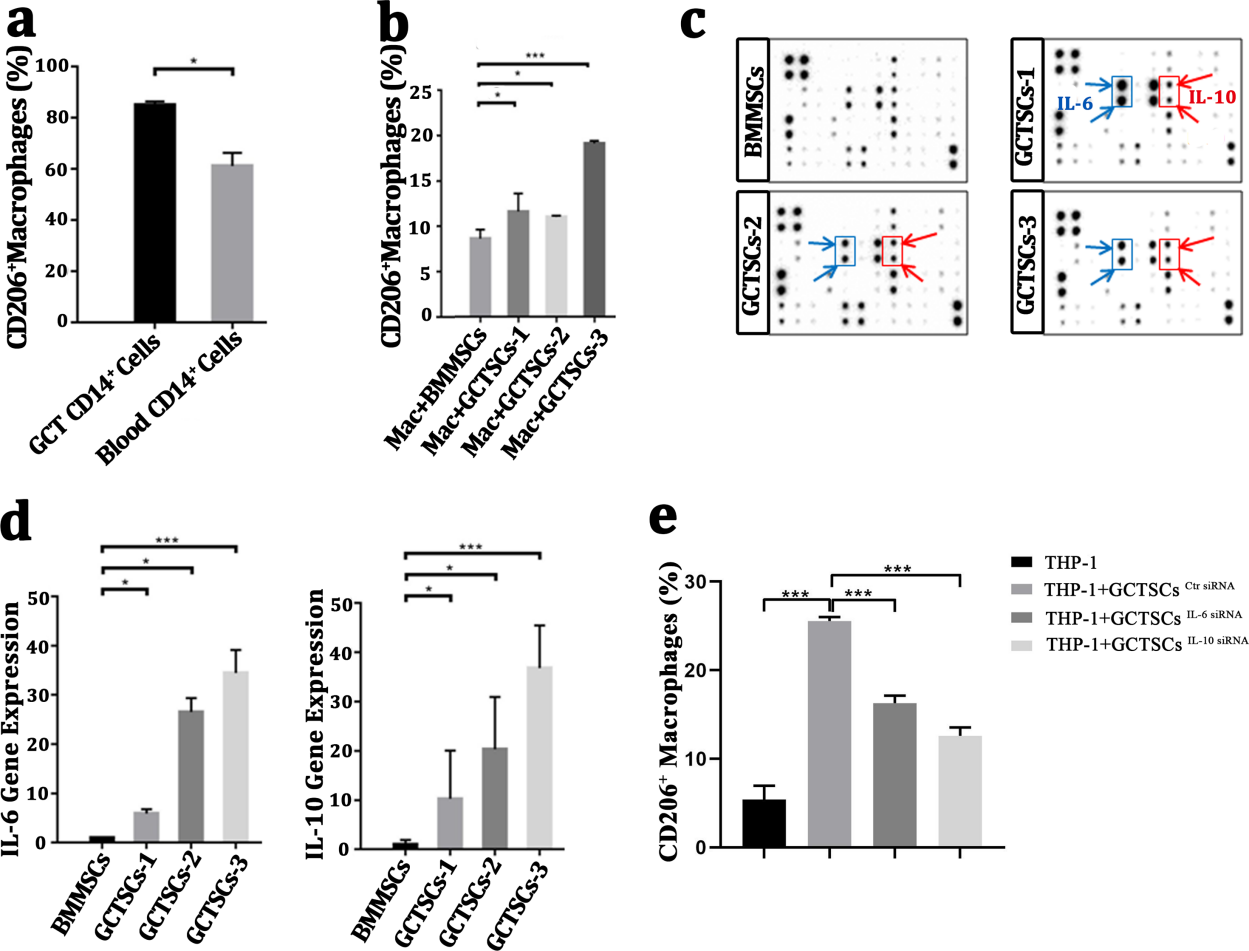
GCTSCs promote the polarization into M2 phenotype of macrophage via increasing IL-6 and IL-10 release. (A) The proportion of CD206^+^ cells among CD14^+^ cells. CD206 is a marker of M2 macrophages and it consists 85.05% of macrophages in GCT compared with 61.15% in blood (*P* < 0.05). (B) CD206 ^+^ cells proportion in GCT CD14^+^ cells and co-cultured with BMMSC, GCTSC1, GCTSC2 and GCTSC3 (GCT CD14^+^ cell vs. GCT CD14^+^ cell +BMMSCs, *P* = 0.483. GCT CD14^+^ cell vs. GCT CD14^+^ cell +GCTSCs1, *P* = 0.026. GCT CD14^+^ cell vs. GCT CD14^+^ cell +GCTSCs2, *P* = 0.044. GCT CD14^+^ cell vs. GCT CD14^+^ cell +GCTSCs3, *P* < 0.001). (C) Human cytokine antibody array analysis of BMMSCs and GCTSCs. Three independent experiments obtained a similar result and one of them are shown. The expression of IL-6 and IL-10 of GCTSCs is much higher compared with BMMSCs (POS=Positive Control Spot, NEG=Negative Control Spot, BLANK=Blank Spot). (D) mRNA expression level of IL-6 and IL-10 among BMMSCs and GCTSCs. The expression of IL-6 and IL-10 of GCTSCs is much higher compared with BMMSCs (^∗^*P* < 0.05, ^∗∗∗^*P* < 0.001). (E) The proportion of CD206^+^ cells among THP-1 cells.

It had been wildly accepted the polarization of macrophage into M2 phenotype was a contribution for tumor propagation and angiogenesis. To investigate the polarization of macrophages in GCT, we detected the proportion of M2 phenotype macrophages in GCT and peripheral blood respectively, we found a numerous proportion of M2 phenotype macrophages among the neoplasm (85.05%) compared with normal macrophages (61.15%) (*P* < 0.05). This suggests that a high number of M2 macrophages exist in giant cell tumor of bone. (Fig. 4A). By co-culture the macrophages with GCTSCs as well as BMMSCs, it aroused a significant (11.0%, 11.6%, 19.1%) polarization of M2 macrophages in GCTSCs and 8.6% in BMMSCs (Fig. 4B). These results verified that, compared with BMMSCs, GCTSCs could promote the macrophages into M2 phenotype.

Human cytokine antibody array can semi-quantitative detected 42 human proteins expression using 2-D densitometry. By analyzing the culture supernatant of BMMSCs and GCTSCs, it was clarified that GCTSCs had a distinctive extraction in the spot of IL-6 and IL-10 (Fig. 4C). q-PCR analysis verified that GCTSCs had a much higher expression of IL-6 and IL-10 compared with BMMSCs (*P* < 0.05) (Fig. 4D). In order to verify the promotion effect of increased GCTSCs IL-6/10 expression on GCT macrophages M2 activation, we used siRNA to reduce the expression of IL-6/10 in GCTSCs, and then co-cultured with THP-1 cells for 48 h. The results of cell flow cytometry showed that knock down the expression of IL-6/10 in GCTSCs could reduce the proportion of THP-1 CD206 positive cells in the co-culture system (*P* < 0.05) (Fig. 4E). These results illustrated that GCTSCs expressed and secreted a large amount of IL-6 and IL-10, which might induce the polarization into M2 phenotype macrophages.

## Discussion

It has been wildly accepted that GCTSCs are the neoplasms component of the bone and express mesenchymal stem cell (MSCs) markers (Werner, 2006; Wülling, Delling & Haiser, 2003; Lan et al., 2012). In this study, we confirm GCTSCs express MSCs-like markers STRO-1, SSEA-4, CD90, CD146, CD105, CD29, which are in accordance with BMMSCs (Lan et al., 2012; Lin et al., 2013), and is the neoplastic component, which have a prominent proliferative activity that can be stained in Brdu measurement. Others reported that GCTSCs expressed alkaline phosphatase, osteocalcin and Cbfa1, equipping a multi-differentiation ability to form mineralized node and adipose not only in vitro, but also in vivo (Murata et al., 2005; Nishimura et al., 2005). In the present results, GCTSCs could differentiate into osteoblasts and adipocytes, which was in accordance with the previous findings. However, comparing with the BMMSCs, GCTSCs have weaker differentiation capacities.

Previous researches had reported that tumor cells could recruiting circulating monocytes, osteoclast precursor cell or even osteoclasts by producing transforming growth factor-1 (TGF- *β*1) and monocyte chemoattractant protein 1 (MCP-1) (Huang et al., 2000). Jenkins SJ et al. discovered that IL-4 and filarial nematode infection can induce resident macrophages proliferation without recruiting blood cells (Jenkins et al., 2011). In order to confirm if GCTSCs can alter macrophages proliferative ability, we co-cultured macrophage cells with GCTSCs and BMMSCs using transwell. The Ki67^+^ cells exhibit a similar proportion among control, GCTSCs and BMMSCs groups, which means GCTSCs don’t induce macrophages proliferation. Simultaneously, the proportion of Annexin V+7ADD+ macrophages was increased after co-culture with BMMSCs, indicating that BMMSCs can induce more macrophage cells apoptosis than GCTSCs.

Nevertheless, no former research has focused on the relationship between GCTSCs and macrophages polarization. We for the first time discovered a numerous proportion of M2 phenotype macrophage cells among the neoplasm (85.05%) compared with normal macrophages (63.15%) (*P* < 0.05). Then, we co-cultured GCTSCs with macrophages and it exhibited a higher proportion of M2 macrophages than BMMSCs. By analyzing the culture supernatant of BMMSCs and GCTSCs with human cytokine antibody array, we found a higher IL-6 and IL-10 secretion, consistent with the array data, an increased mRNA expression was found in IL-6 and IL-10. Interestingly, knockdown of the IL-6 and IL-10 expression in GCTSCs cells with siRNA attenuated its effect on THP-1 cells M2 polarization. Therefore, we conclude that the cytokines secreted by GCTSCs may engage in macrophage M2 polarization.

It has been clarified that MSCs can alter the phenotype of macrophages from M1 type into a M2 population, which are indicative of pro-tumor activity (Ohashi et al., 2018; Hajkova et al., 2017), but there are no articles that analyze macrophage phenotype among GCT. Former researchers had claimed that immunoregulation cytokines like IL-4, IL-10 and IL-13 induced the M2 phenotype polarization (Goswami et al., 2017). However fewer articles reported that IL-6 may also be a novel cytokine that plays an important role in M2 macrophage polarization (Mauer et al., 2014; Fu et al., 2017). In our research, IL-6 is obviously involved in the polarization of macrophages M2, but the mechanism still needs more exploration. STAT3 is an important mechanism for inducing the activation of tumor-associated macrophages M2 type (Yin et al., 2018). It has been reported that in the tumor microenvironment, the IL-6/STAT3 signaling pathway plays an important role in the macrophage M2 polarization, and activation of the IL-6/STAT3 signaling pathway causes macrophages to polarize towards the M2 phenotype. Inhibiting the IL-6/STAT3 signaling pathway correspondingly shifts macrophages to the M1 polarization phenotype, and blocking the IL-6/STAT3 signaling pathway can dramatically reduce tumor formation (Yin et al., 2018). Further, IL-6 binds to the IL-6 receptor subunitα (IL-6RA) and the co-receptor gp130 to activate JAK and signal transduction and transcription activator 3 (STAT3) phosphorylation, thereby activating the inflammatory cascade (Yin et al., 2018). In addition, IL-10 can induce IL-4Rα expression and increase Arg1 expression, the one of markers of macrophage M2 activation. It was also be reported that IL-10 can enhance the expression of M2a macrophage-related genes in M2 macrophages induced by IL-4 in vitro (Mu et al., 2018). As we have detected both IL-6 and IL-10 high secretion of GCTSCs, it’s interested for us to explore the connection between GCTSCs and M2 macrophages. In this article, there are still many phenomena need further explore. (a) Whether the M2 polarization on account of GCTSCs play a significant role in GCT invasion and metastasis. (b) The exact mechanisms of IL-6 and IL-10 in inducing M2 polarization among GCT are still unknown. In conclusion, we first confirm that GCTSCs are belong to osteoblast lineage and have an enhanced proliferative ability as well as a deduced differentiative ability. Although numerous populations of macrophages emerge in giant cell tumor, GCTSCs have none influence on its proliferation of apoptosis. IL-6 and IL-10 are found expressed highly among GCTSCs, while they are speculated to play a significant role in M2 phenotype macrophages. Our findings are consistent with previous reports that high level of M2 phenotype activation of tumor-associated macrophages in tumor microenvironment. In particular, we found that the increased expression of GCTSCs IL-6 and IL-10 is the key factor that causes the activation of M2 of macrophages. This provides a novel prospect for the clinical treatment of giant cell tumors of bone.

**Figure 5:**
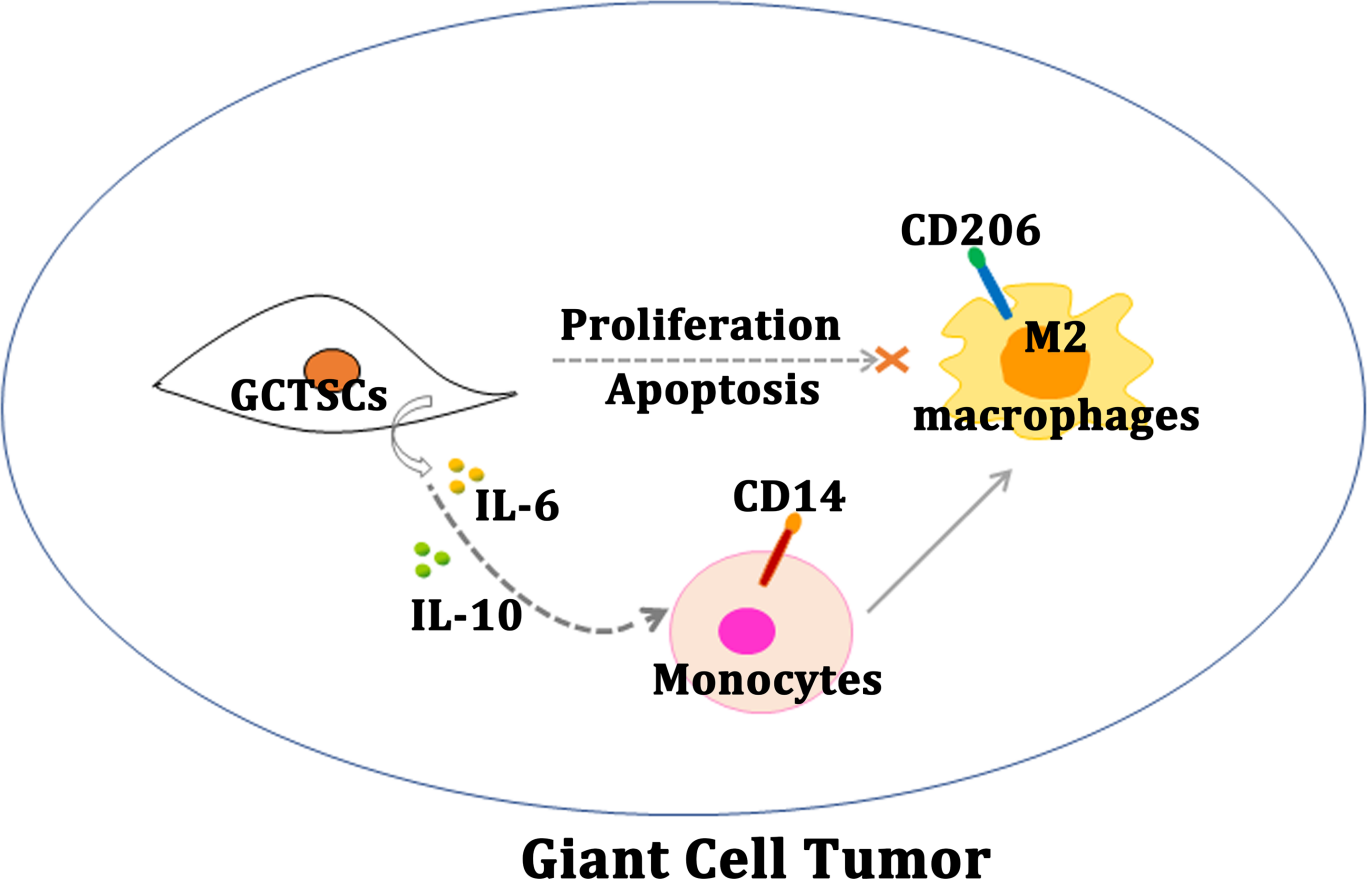
Schematic diagram of this study. Schematic illustration of GCTSCs exist in giant cell tumor, and release IL-6 and IL-10 to promote macrophages turning into M2 polarization phenotype.

## Conclusions

Our research proves that, GCTSCs expressed MSCs markers and had higher proliferation and relative lower differentiation abilities compared with BMMSCs. Co-culturing GCTSCs with macrophages promoting macrophages polarization into M2 phenotype (Fig. 5). At the same time, the level of IL-6 and IL-10 released from GCTSCs was increased. In short GCTSCs contribution for tumor proliferation via promoting macrophages M2 phenotype polarization by improving IL-6 and IL-10 secretion (Fig. 5).

##  Supplemental Information

10.7717/peerj.9748/supp-1 Supplemental Information 1 Numeric data Figs. 2B and 2C: The raw data of fluorescence intensity of sections labeled with fluorescence analyzed by image J. Fig. 2D: The raw data of dyeing strength of alizarin red analyzed by image J. Fig. 2E: The raw data of dyeing strength of oil red O analyzed by image J. Fig. 2F: The raw data of dyeing strength of alizarin red analyzed by image J. Fig. 3A: The raw data of the number of macrophages detected by cell flow cytometry. Figs. 3B and 3C: The raw data of Ki 67 and Annexin V+7ADD positive cell percentage using flow cytometry. Figs. 4A and 4B: The raw data of the percentage of CD206 positive cells detected by cell flow cytometry. Fig. 4D: The raw data of expression level of IL-6 and IL-10 detected by quantitative RT-PCR. Fig. 4E: The raw data of CD206 positive cell percentage using flow cytometry.

## Additional Information and Declarations

### Data Availability

The following information was supplied regarding data availability: The raw data is available as a Supplementary File.
